# Molecular mechanism of acquisition of the cholera toxin genes

**Published:** 2011-02

**Authors:** Bhabatosh Das, Julien Bischerour, François-Xavier Barre

**Affiliations:** *CNRS, Centre de Génétique Moléculaire, Gif-sur-Yvette & Université Paris-Sud, Orsay, France*

**Keywords:** *dif*, site-specific recombination, XerC, XerD

## Abstract

One of the major pathogenic determinants of *Vibrio cholerae*, the cholera toxin, is encoded in the genome of a filamentous phage, CTXφ. CTXφ makes use of the chromosome dimer resolution system of *V. cholerae* to integrate its single stranded genome into one, the other, or both *V. cholerae* chromosomes. Here, we review current knowledge about this smart integration process.

## Introduction

Most bacteriophages are detrimental to their host metabolism. However, phages also participate in the horizontal transfer of genes among bacteria because their genome can harbour other genes than those strictly required for their life cycle. This can be highly beneficial to the bacterial host. Indeed, many bacterial virulence factors are associated with phage-like DNA sequences. More strikingly, the exotoxins produced by many pathogenic bacteria are encoded in the genome of lysogenic phages. This is notably the case in *Bordetella avium^1^, Clostridium botulinum^2^, Corynebacterium diphtheria^3^, *Escherichia coli*^4^, Pseudomonas aeruginosa^5^, Shigella dysenteriae^6^, Staphylococcus aureus^7^ and Streptococcus pyogenes*^8^. The integrated prophages harboured by these bacteria profit from the multiplication of their host in the environment, which is in turn favoured by the virulence factors they bring to their host.

The study of *Vibrio cholerae*, the agent of the deadly diarrhoeal disease cholera, provides a fascinating case of such a bacterium-phage co-evolution. *V. cholerae* is the host for a variety of phages, commonly known as vibriophages, which can be lytic, non-lytic, virulent or temperate^9^. On the one hand, phage predation of *V. cholerae* has been reported to be a factor that influences seasonal epidemics of cholera^10^. On the other hand, one of the major virulence factors of *V. cholerae*, cholera toxin, is encoded in the genome of an integrated prophage CTXΦ^11^,^12^. Furthermore, different variants of the phage CTXΦ exist, which participate in the genetic diversity of epidemic causing cholera strains^13^–^15^. Two different attachment sites were found for this family of phages on the *V. cholerae* genome. They correspond to the dimer resolution sites of the two *V. cholerae* chromosomes, *dif*1 and *dif*2^16^. Indeed, in contrast to most other lysogenic phages, such as bacteriophage λ^17^, CTXΦ does not encode its integrase, but makes use of XerC and XerD, the two host-encoded tyrosine recombinases that normally function to resolve chromosome dimmers^18^. This mode of integration is all the more intriguing since CTXΦ phages belong to the filamentous phage family, which are generally not lysogenic and which harbour a single stranded circular genome. Nevertheless, CTXΦ-like prophages were found integrated in the genome of several bacterial species, notably in pathogenic *E. coli* strains^19^ and in *Yersinia pestis*^20^. Finally, it is remarkable to observe that many filamentous phages and/or genetic elements other than CTXΦ seem to have hijacked the chromosome dimer resolution system of *V. cholerae* for integration. Thus, TLC^21^, VEJ^22^, VGJ^23^, VSK^24^, VSKK (AF452449), KSF-1F^24^, fs1^25^, fs2^26^, f237^14^, were all found to be integrated at *dif*1 and/or *dif*2. Such a diversity of elements has not been observed in any other genera than the vibrios. Together, these elements participate in the dissemination of virulence factors among *V. cholerae* strains^11^,^28^,^29^ and in the emergence of new genetic variants of epidemic strains of *V. cholera*^13^. We review current knowledge on the integration mechanism of filamentous vibriophages that hijack the XerCD recombinases, with a special focus on CTXΦ.

## CTXΦintegration mechanism: exception or new paradigm?

CTXΦ has a ~7-kb ss(+)DNA genome arranged in two modular structures, the “RS” and“core”. The core region harbours seven genes, which are *psh, cep, gIIICTX, ace, zot, ctxA* and *ctxB*. While the *psh, cep, gIIICTX, ace* and *zot* encoded proteins are needed for phage morphogenesis, the products of the *ctxAB* genes are not strictly required for the life cycle of the phage but are responsible for the severe diarrhoea associated with cholera^11^. Three proteins, designated as RstR, RstA and RstB, are encoded in RS. Genetic analyses indicated that RstA is essential for phage replication and that RstB plays a crucial role in integration^30^. RstR acts as a transcriptional repressor by inhibiting the activity of P_rstA_, the only phage promoter required for CTXΦ replication and integration^30^. Several CTXΦ have been reported. These can be classified into four families based on the sequence of their *rstR* gene. These categories were designated as CTXΦ^ET^, CTXΦ^Cl^, CTXΦ^Clc^ and CTXΦ^Env^ according to the host cells in which they were originally isolated^31^–^33^.

As mentioned earlier, the integration of CTXΦ into the *V. cholerae* genome depends on two host encoded tyrosine recombinases, XerC and XerD^18^. XerC and XerD normally serve to resolve circular bacterial chromosome dimers generated by RecA mediated homologous recombination by adding a crossover at a specific 28 bp site *dif* on the chromosome^16^. The *dif* sites consist of specific 11-bp binding sites for each of the two Xer recombinases, separated by a 6-bp central region^34^. These are generally located opposite to the origin of replication of bacterial chromosomes^16^. Two *dif* sites are present on the genome of *V. cholerae*, one for each of the two circular chromosomes of the bacterium^35^. Three different chromosome dimer resolution sites (*dif*1, *dif*2 and *dif*G) have been identified among the different *V. cholerae* strains characterized to date^36^ (Table I).

**Table I:** Sequences of the chromosome dimer resolution sites found in *V. cholerae* strains

Site	Sequence
*dif*1	AGTGCGTATTA TGTATG TTATGTTAAAT
*dif*2	AATGCGTATTA CGTGCG TTATGTTAAAT
*difG*	AGTGCGTATTA GGTATA TTATGTTAAAT
*Source*: Ref. 36	

**Fig. 1:**
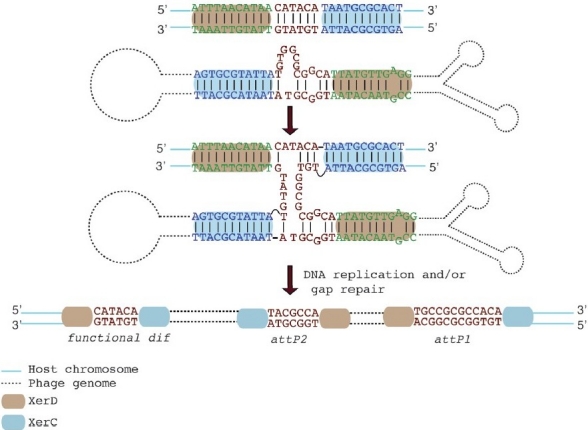
Schematic representation of the XerCD mediated site-specific recombination reaction between the single stranded (+) DNA genome of CTXΦ and *V. cholerae* *dif*1. Blue and green bases indicate XerC and XerD binding sites. Bases of the central region of these sites are shown in red. The recombination reaction stops after the exchange of a single pair of strands, which is catalyzed by XerC. Integration is completed when the resulting pseudo-Holliday junction needs to be processed by the host DNA replication and/or DNA repair machineries. Integration of the phage generates one new functional *dif* site and two non-functional *dif* like sequences, *attP2* and *attP1*, on the host chromosome^38^.

The ssDNA (+) genome of CTXΦ harbours two *dif* like sites (*attP1* and *attP2*). These are arranged in opposite orientation and are separated by ~90-bp DNA segment in the phage genome^37^. Integration of CTXΦ at the *dif* loci of *V. cholerae* depends on the formation of a forked hairpin structure of 150 bp in the region encompassing *attP1* and *attP2* in the (+) ssDNA genome^38^ (Fig. 1). The hybridization of *attP1* and *attP2* at the stem of this hairpin unmasks the phage attachment site, *attP*(+). Integration occurs, XerC and XerD recombine this site with one of the two dimer resolution sites harboured by the host cell. This process only requires the catalytic activity of XerC: a single pair of strands is exchanged, which results in the formation of a pseudo-Holliday junction.

A proof of principle for this mechanism of integration was originally obtained for the El Tor variant of CTXΦ and *dif*1 based on *in vivo* work performed in *Escherichia coli* and *in vitro* work performed with the *E. coli* Xer recombinases^38^. Later on, a sensitive and quantitative assay was developed to confirm the ssDNA(+) integration model of CTXΦ^ET^ into the *dif*1 site of a *V. cholerae* El Tor strain^36^. This system was also used to define rules of compatibilities between the phage attachment sites harboured by the different CTXΦ variants characterized to date and their host dimer resolution sites^36^ : integration is solely determined by possibility to form Watson-Crick or w0 obble base pair interactions to stabilize the exchange of strands promoted by XerC-catalysis between the phage attachment site and its target dimer resolution site (Table II and Fig. 1). These rules explain how integration of CTXΦ^ET^ is restricted to *dif*1, how CTXΦ^Cl^ can target both *dif*1 and *dif*2, and how a third CTXΦ variant targets *dif*G (Table II). This single stranded integration model is not restricted to CTXΦ. Analysis of the *att* P sites of CUS-1F and Ypf-F phages revealed features for direct ssDNA integration into the chromosome dimer resolution site harboured by their respective host cells^38^. Another family of mobile genetic element, the integrons, also integrates in the bacterial chromosome via a single stranded intermediate^39^.

**Table II:** Sequences of the *dif*-like sites harboured by CTXΦ variant

CTXΦ variant	*attP* sequence	Integration site	Accession number
El Tor	AGTGCGTATTA TGTGGCGCGGCA TTATGTTGAGG *(attP1)* AATGCGTATTA TACGCCA TTATGTTACGG *(attP2)*	*dif*1	VCU83796
Classical	AGTGCGTATTA TGTGGCGCGGCA TTATGTTGAGG *(attP1)* AATGCGTATTA CTCGCCA TTATGTTACGG *(attP2)*	*dif*1 dif2	AY349175
Calcutta	AGTGCGTATTA TGTGGCGCGGCA TTATGTTGAGG *(attP1)* AATGCGTATTA TACGCCA TTATGTTACGG *(attP2)*	*dif*1	AF110029
G	AGTGCGTATTA GGTGGTGCGGCA TTATGTTGAGG *(attP1)* AATGCGTATTA GGGGCA TTATGTTACGG *(attP2)*	*dif*G	AF416590
*Source*: Ref. 40			

## Integration mechanism of CTXΦ-associated genetics elements

**Fig. 2:**
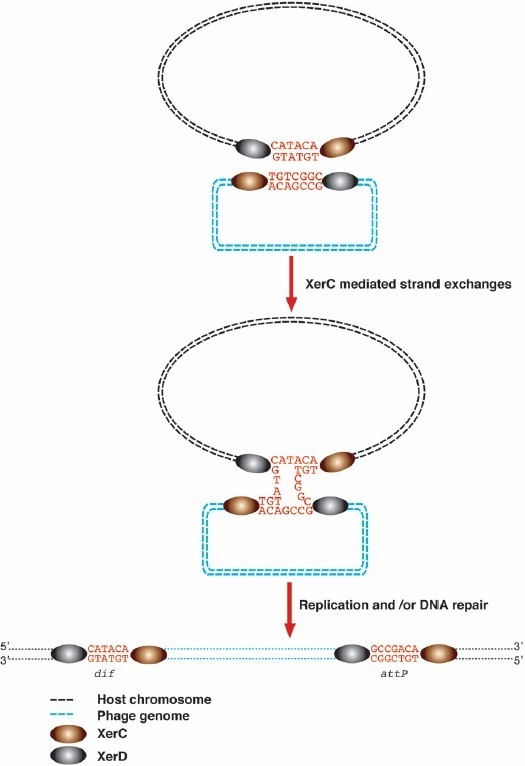
Putative mechanism of lysogenic conversion by the second type of filamentous phages that are found integrated into the chromosomal dimer resolution sites of *V. cholerae*^40^.

Several filamentous phages other than CTXΦ are found to be integrated at the *dif* loci of *V. cholera*^13^,^22^,^23^. To date, there is no report about their particular integration mechanism. Like CTXΦ, they do not encode a dedicated recombinase. In addition, a 29-bp *dif* like sequence can be identified in many of them (Table III). It is, therefore, very likely that these phages take control of the host XerC and XerD recombinases to integrate into the genome of their host. However, the presence of a single putative XerCD binding site on their genome makes it unlikely that the ssDNA form of their genome is directly used as a substrate for integration. We rather favour a model in which the double stranded replicative form of these phages is used for integration (Fig. 2). We are currently investigating this model using the tools we have developed for the study of CTXΦ^40^.

**Table III:** Sequences of the *dif-*ured by other vibriophages

Phage	Genome size (kb)	*attP* sequence	Host	Integration site	Accession number
VEJ	6.8	ACTTCGCATTA TGTCGGC TTATGGTAAAA	*V. cholerae*	*dif1*	NC012757
VGJ	7.5	ACTTCGCATTA TGTCGGC TTATGGTAAAA	*V. cholerae*	*dif1*	AY242528.1
VSK	6.9	ACTTCGCAGTA TGTCGGC TTATGGTAAAA	*V. cholerae*	*dif1*	NC003327
VSKK	6.8	ACTTCGCATTA TGTCGGC TTATGGTAAAA	*V. cholerae*	*dif1*	AF452449
KSF1	7.1	UK	*V. cholerae*	UK	AY714348
fs1	6.3	UK	*V. cholerae*	UK	NC004306.1
fs2	8.6	AGTGCGTATTA TGTCGGC TTATGGTAAAA	*V. cholerae*	*dif1*	AB002632
f237	8.7	AGTGCGCATTA TGGGCGC TTATGTTGAAT	*V. cholerae V. parahemolyticus*	*dif1*	NC002362
UK, unknow; *Source*: Ref. 40					

Interestingly, the two TLC elements integrated in strain N16961 are flanked by the half of the *dif* sequence (TGTGCGCATTA TGTATG for one and AGTGCATATTA TGTATG for the other). It is, therefore, reasonable to argue that their integration might be linked to the activity of the Xer recombinases.

## Future prospects

The particular mode of integration of CTXΦ raises several questions. First, the efficiency of integration of a circular single stranded DNA molecule harbouring the sole attachment site of CTXΦ is very low^38^. However, it becomes extremely efficient when the RS region of the phage is included^36^. One likely explanation is that constant production and/or stabilization of the phage single stranded circular genome compensate for the instability of single stranded DNA in bacterial cells. RstB, which has been shown to be a single stranded DNA binding protein^41^, could play a role in the stabilization of the integration substrate. Accordingly, its biochemical properties and sequence differ from those of the single stranded DNA binding protein encoded in the genome of VGJF, a phage that seems to rely on double stranded DNA integration^40^. Second, only one pair of strands is exchanged between the single stranded DNA genome of CTXΦand the double stranded DNA genome of its host, which leaves open the question of how the resulting pseudo-Holliday junction intermediate is processed. Is it stably maintained until the next round of bacterial DNA replication or processed by some host DNA repair machinery? What occurs when the replication fork collides against this unusual structure? Finally, it is intriguing that so many phages take advantage of the Xer recombination system of vibrios as compared to other bacterial species. We wonder if it could be related to the particular life style and environment of the vibrios and/or their particular genome structure and management.
